# Lorazepam causing drug-induced liver injury : Rare entity

**DOI:** 10.1192/j.eurpsy.2021.2046

**Published:** 2021-08-13

**Authors:** S. Sellami, H. Mami, M. Moalla, M. Oumaya, R. Bouzid

**Affiliations:** 1 Department Of Psychiatry, University hospital of Nabeul, Nabeul, Tunisia; 2 Department Of Gastroenterology, University hospital of Nabeul, Nabeul, Tunisia

**Keywords:** Lorazepam, DILI, Liver Injury, drug

## Abstract

**Introduction:**

Lorazepam is a benzodiazepine derivative that is globally used for the therapy of anxiety and insomnia.

**Objectives:**

The objective of our work was to show that Lorazepam can be a cause of unexpected liver injury even though it is a rare entity.

**Methods:**

We reported the case of a patient who had a Drug-Induced Liver Injury (DILI) under Lorazepam. We performed a literature review based on a PubMed search with the following keywords: “Lorazepam,DILI”.

**Results:**

A 20 year-old-Tunisian woman was hospitalized in the psychiatry department of the hospital of Nabeul in Tunisia for a brief psychotic episode.She had a DILI under Olanzapine, Chlorpromazine and Lorazepam, which conducted us to interrupt her treatments except for the Lorazepam(5mg/day). The hepatic tests went back to normal even under Lorazepam. Few days later, the liver enzymes increased again to reach very high levels. Extensive workup was negative for other causes of liver injury, including viral hepatitis A, B, C and E.; capillary electrophoresis of serum proteins was normal; Exhaustive immunological tests were performed searching for auto immune hepatitis(anti-smooth muscle antibodies, anti-LKM1, anti-LC1, anti-SLA/LP) primary biliary cholangitis(anti-mitochondrial antibodies, anti-GP210, anti-sp100) and other antibodies like antinuclear antibodies were negative. Liver biopsy showed polymorphic inflammatory infiltrate including some eosinophilic polynuclear cells and rare vaguely epitheloid macrophages, with necrotico-inflammatory foci in the lobules, all of which were consistent with DILI. Lorazepam was discontinued and within 10 days her liver enzymes decreased and completely normalized.

**Conclusions:**

Lorazepam, with an unknown action mechanism, can be a cause of DILI.

**Disclosure:**

No significant relationships.

